# Prognostic value of Beclin 1, EGFR and ALK in non-squamous non-small cell lung cancer

**DOI:** 10.1007/s12672-022-00586-y

**Published:** 2022-11-19

**Authors:** Yanhui Wan, Youhui Qian, Youyu Wang, Fuyuan Fang, Guodong Wu

**Affiliations:** Department of Thoracic Surgery, the First Affiliated Hospital of Shenzhen University/Shenzhen Second People’s Hospital, 3002 Futian Road , Shenzhen, 518000 China

**Keywords:** Prognosis, Beclin 1, EGFR, ALK, Non-squamous NSCLC

## Abstract

Non-small cell lung cancer (NSCLC) is one of the most malignant tumors. The study was carried out to investigate the prognostic value of Beclin 1, EGFR and ALK for this cancer. Patients diagnosed with non-squamous NSCLC and admitted to our hospital from January 2011 to September 2016 were analyzed. Expression of Beclin 1 and mutation of EGFR and ALK were assessed using polymerase chain reaction (PCR) and fluorescent in situ hybridization (FISH) and analyzed for their relationship with demographic and clinical characteristics of the patients. Multivariate Cox regression models were applied to analyze the risk factors associated with survival and receiver response curves (ROC) were plotted to determine the prognostic value of Beclin 1, EGFR and ALK for patients with non-squamous NSCLC. Compared with adjacent normal tissue, Beclin 1 expression was elevated in the cancer tissue significantly; assessments of EGFR and ALK mutations showed that out of the 480 patients, 233 (48.5%) and 75 (12.6%) patients had EGFR and ALK mutations. Univariate analysis revealed that Beclin 1 level, EGFR and ALK mutations were associated with lymph node metastasis, TNM stage, tumor differentiation and prognosis, but not with gender, age and smoking status. The Kaplan–Meier survival analysis indicated that low Beclin 1 expression and positive EGFR and ALK rearrangements were associated with higher survival rate and longer progress-free survival (PFS). Multivariate Cox regression analysis showed that Beclin 1, EGFR, ALK mutations, tumor differentiation grade, TNM stage and lymph node metastasis were independently associated with PFS. ROC analysis showed that Beclin 1, EGFR and ALK were significant predictors for PFS; the areas under curve (AUC) for Beclin 1, EGFR and ALK were 0.812 (*P* = 0.018, cut-off value: 1.2), 0.781 (*P* = 0.011, cut-off value: 15%) and 0.722 (*P* = 0.010, cut-off value: 11%), respectively, suggesting that they have significant prognostic value for lung cancer patients. Our data indicate that Beclin 1, EGFR and ALK genes are associated with the prognosis of patients with non-squamous NSCLC. High Beclin 1 expression and negative EGFR and ALK mutations predict a poor prognosis with PFS.

## Introduction

Lung cancer is a common malignant tumor which seriously endangers people's health and life. The incidence and mortality rate of the cancer rank the first among the cancers in the world [1]. Even if patients with early lung cancer have undergone surgical resection, their clinical prognosis is still very poor with only 53% 5-year survival rate and as high as 40% 5-year recurrence rate [2, 3]. Therefore, reducing the distant recurrence of early lung cancer and the death caused by recurrence is of great significance to improve the prognosis of lung cancer patients.

With the rapid development of precision cancer medicine, a better understanding of lung cancer-related gene mutations has allowed to detect mutations in genes such as EGFR, ALK, BRAF, HER2, met, ros1 and RET to stratify patients for better treatments, leading to increased survival time of lung cancer patients after targeted treatments [4–6]. In recent years, predicting postoperative mortality of early lung cancer by analyzing tumor-related gene expression has gradually become a focus of attention [7–11].

The prevalence of early-stage non–small cell lung cancer (NSCLC) is expected to increase with recent implementation of annual screening programs. According to histological typing, 55% of NSCLC belong to lung adenocarcinoma, 34% are squamous cell carcinoma and 11% are others [12]. Clinically, NSCLC is generally divided into squamous and non-squamous NSCLC to better guide postoperative adjuvant chemotherapy. Although a number of prognostic biomarkers have been developed for NSCLC, including immunohistochemical markers [13, 14], protein markers [15, 16] and miRNA markers [17, 18] for targeted therapy and immune therapy, there is still a lack of methods and means to identify patients with high risk of recurrence to guide adjuvant therapy in patients with non-squamous NSCLC.

Previously studies have demonstrated that the dysfunction of Beclin 1, a major regulator of autophagy and a core component of the class III PI3K complexes [19, 20], may lead to diseases as well as cancer [21]. It is demonstrated to have significant prognostic and clinicopathological significance in cancers, such as NSCLC [22], gallbladder cancer [23] oral cancer [24].The epidermal growth factor receptor (EGFR) belongs to the ERBB family of tyrosine kinase receptors. It has been identified as an oncogenic driver of NSCLC [25], and mutations in EGFR often result in the activation of pathways leading to cell growth, DNA synthesis and the expression of oncogenes [26]. The anaplastic lymphoma kinase (ALK) gene encodes a transmembrane tyrosine kinase receptor. It has been associated with many types of cancers, including NSCLC [27]. Screening for *ALK* rearrangements has become a very important process in treatment decision making for advanced NSCLC [28] and mutation tests of these genes, including their rearrangements, are part of cancer gene therapy strategies for NSCLC patients [29]. However, whether these genes have prognostic value for patients with non-squamous NSCLC is not fully clear.

In the present study, we analyzed the expression and mutation of Beclin 1, EGFR and ALK in patients with non-squamous NSCLC and their relationship with postoperative survival. The findings would provide new prognostic markers for better management of the disease.

## Materials and methods

### Patients

This is a retrospective study conducted at the First Affiliated Hospital of Shenzhen University, China. Consecutive elderly patients admitted to our hospital from January 2011 to September 2016 were included in the study. Patients were included if he/she was older than 65 years, diagnosed pathologically based on histological assessments and radiologically to have non-squamous NSCLC without distant metastasis in the first diagnosis, complied with treatment protocols and had complete clinical data. Patients were excluded if they had other primary malignant tumors or tumor metastasis, or had dysfunction in important organs such as heart failure (left ventricular pressure > 10 mmHg in systolic heart failure, left ventricular pressure > 5 mmHg in diastolic heart failure), kidney failure (estimated glomerular filtration rate < 15) and liver failure (based on blood tests), or had cerebrovascular diseases.

This study was approved by the ethical committee of Shenzhen University and written informed consent was obtained from every patient.

### Data collection

Demographic and clinical data were collected from the hospital data bases, including gender, age, pathological type, clinical stage and survival.

### Quantitative reverse transcription-polymerase chain reaction (qRT-PCR)

The expression of Beclin 1 was detected using qRT-PCR. Total RNA was extracted from the tissue samples taken from all enrolled patients for pathological examination using RNeasy Total RNA Kit (Qiagen, USA) according to manufacturer’s instructions. Extracted RNA was quantified using a Nanodrop spectrophotometer (NanoDrop Technologies, USA) and reversely transcripted into cDNA using the High-Capacity cDNA Transcriptase Reverse kit (Applied Biosystems by Life Technologies, Carlsbad, California, USA) according to the manufacturer’s recommendations. qRT-PCR was carried out using Universal SYBR qPCR Master Mix (Applied Biosystems by Life Technologies, Carlsbad, California, USA) on an Applied Bio-Rad CFX96 instrument. Primers PCR are listed in Table 1. The relative mRNA levels were determined using the 2^−ΔΔCt^ method after normalization with β-actin as internal reference [30]. The PCR was carried out in a total volume of 15 μl containing 1 μl of diluted and pre-amplified cDNA, 10 μl of TaqMan Gene Expression Master Mix and 1.5 μl of each fluorescence TaqMan probe. The cycling conditions were 95 ºC for 10 min followed by 40 cycles, each one consisting of 15 s at 95 ºC and 1 min at 57 ºC.

**Table 1:** Primers for PCR

Genes	Primer sequences
Beclin (forward)	5′-TGGAGGGCAGTCCATACCCTGG
Beclin (reverse)	5′-GAGCTGGCTCCTGTGAGTATG
β-actin (forward)	5′-GCACCACACCTTCTACAATG
β-actin (reverse)	5′-TGCTTGCTGATCCACATCTG
EGFR external control (forward)	5′-TGGAGAGCATCCAGT
EGFR external control (reverse)	5′-TCTGGAAGTCCATCGACAT
Del (1) (forward)	5′-CCGTCGCTATCAAA
Del (1) (reverse)	5′-GTCGCTATCAAGA
Del (2) (forward)	5′-GTCGCTATCAAGA
Del (2) (reverse)	5′-GTCGCTATCAAGA

For EGFR, amplification refractory mutation system (ARMS) was used as previous described [31]. Briefly, formalin-fixed paraffin-embedded (FFPE) tumor samples prepared from all enrolled patients for pathological examination were micro-dissected by a pathologist. Genomic DNA was extracted using the QIAamp^®^ DNA FFPE Tissue kit (Qiagen, Shanghai, China) the manufacturer’s protocols. The isolated DNA samples were amplified using EGFR Gene Mutation Quantitative Detection kit (GenoSaber, Shanghai, China) according to the manufacturer's instructions. The kit detecting EGFR mutations in exon 4, 19 20 and 21 was used in this study. 5 µl samples were added to the pre-mixed 45 µl reaction mixtures and the PCR was conducted on Applied Biosystems® 7500 Real-Time PCR Systems (Thermo Fisher Scientific, MA, USA). The PCR reactions were run as follows: hot start at 95 °C for 5 min, followed by 50 cycles of 95 °C for 10 s, 61 °C for 30 s, using primers listed in Table 1. The mutant percentage was calculated as the percentage of mutant copy over total copy present in the sample. The PCR products were detected using a QX-200 droplet reader (Bio-Rad Laboratories) and the data were analyzed using QuantaSoft software (Bio-Rad Laboratories) to calculates the copy number of both mutant and wild-type DNA according to the Poisson statistic.

### Fluorescent in situ hybridization (FISH)

ALK rearrangements were detected using FISH as reported previously [32]. Briefly, FFPE tumor sections prepared from all enrolled patients were rehydrated by going through an ethanol serial and hybridized to fluorescent probe at room temperature for 2 h according to the supplier’s instructions. The probe was purchased from Beyetime, Beijing. FISH signals were analyzed using a fluorescence microscope (Olympus BX51, Tokyo, Japan) equipped with a DP72 camera and DP2-BSW software (Olympus, Tokyo, Japan). 100 nuclei were examined and test results were categorized as negative if < 15 cells (< 15%) had positive signals, positive if > 15 cells (> 15%) had positive signals. The signal distributions were evaluated by two independent observers who were blinded to the patient information.

### Statistical analysis

The data were analyzed using SPSS (version 11.5) for Windows (SPSS Inc., Chicago, IL, USA). The normality of distribution of continuous variables was tested by one-sample Kolmogorov–Smirnov test. Continuous variables with normal distribution were presented as mean (standard deviation (SD)); non-normal variables were reported as median (interquartile range [IQR]). Means of 2 continuous normally distributed variables were compared by independent samples Student's t test. Mann–Whitney U test and Kruskal–Wallis test were used, respectively, to compare means of 2 and 3 or more groups of variables not normally distributed. The frequencies of categorical variables were compared using Pearson χ^2^ or Fisher's exact test, when appropriate. Survival curves were calculated using the Kaplan–Meier method and compared by the log-rank test according to univariate analysis. Receiver operating characteristic (ROC) curves were calculated to predict PFS. Odds ratios (OR) with 95% confidence intervals (CI) were reported. A value of *P* < 0.05 was considered significant.

## Results

### Patient characteristics

A total of 480 patients were enrolled in the five-year study period, including 265 (55.2%) males and 215 (44.8%) females. The median age was 72.5 (70.2, 74.3) and there were 145 (30.2%) smokers. Lymph node metastasis was observed in 321 (66.9%) patients and TNM stage ranged from I–II (231, 48.1%) and III–IV (249, 51.9%). By the end of study, 345 (71.9%) patients survived and 135 (28.1%) patients died (Table 2).

**Table 2:** Relationship between pathological characteristics of non-squamous non-small cell lung cancer patients and Beclin 1 expression, EGFR and ALK mutations

Characteristics		High Beclin 1	*P*	EGFR^+^	*P*	ALK^+^	*P*
No. patients	480	235		233		75	
Age (years), n			0.750		0.650		0.550
≥ 70	254	123		126		45	
60–69	226	112		107		30	
Gender, n			0.218		0.246		0.234
Male	265	124		125		44	
Female	215	111		108		31	
Smoking, n			0.296		0.117		0.423
Yes	145	123		98		32	
No	335	117		135		43	
Lymph node metastasis, n			0.009		0.010		0.010
Yes	321	155		68		9	
No	159	80		165		66	
TNM stage, n			0.050		0.015		0.013
I–II	231	22		155		70	
III–IV	249	213		78		5	
Tumor differentiation, n			0.009		0.032		0.043
Low-grade	112	22		144		40	
Intermediate-grade	167	44		56		23	
High-grade	201	169		33		12	
Prognosis, n			0.019		0.015		0.011
Survival	345	156		198		67	
Death	135	79		35		8	

### Beclin 1 expression, EGFR and ALK mutations

**Fig. 1:**
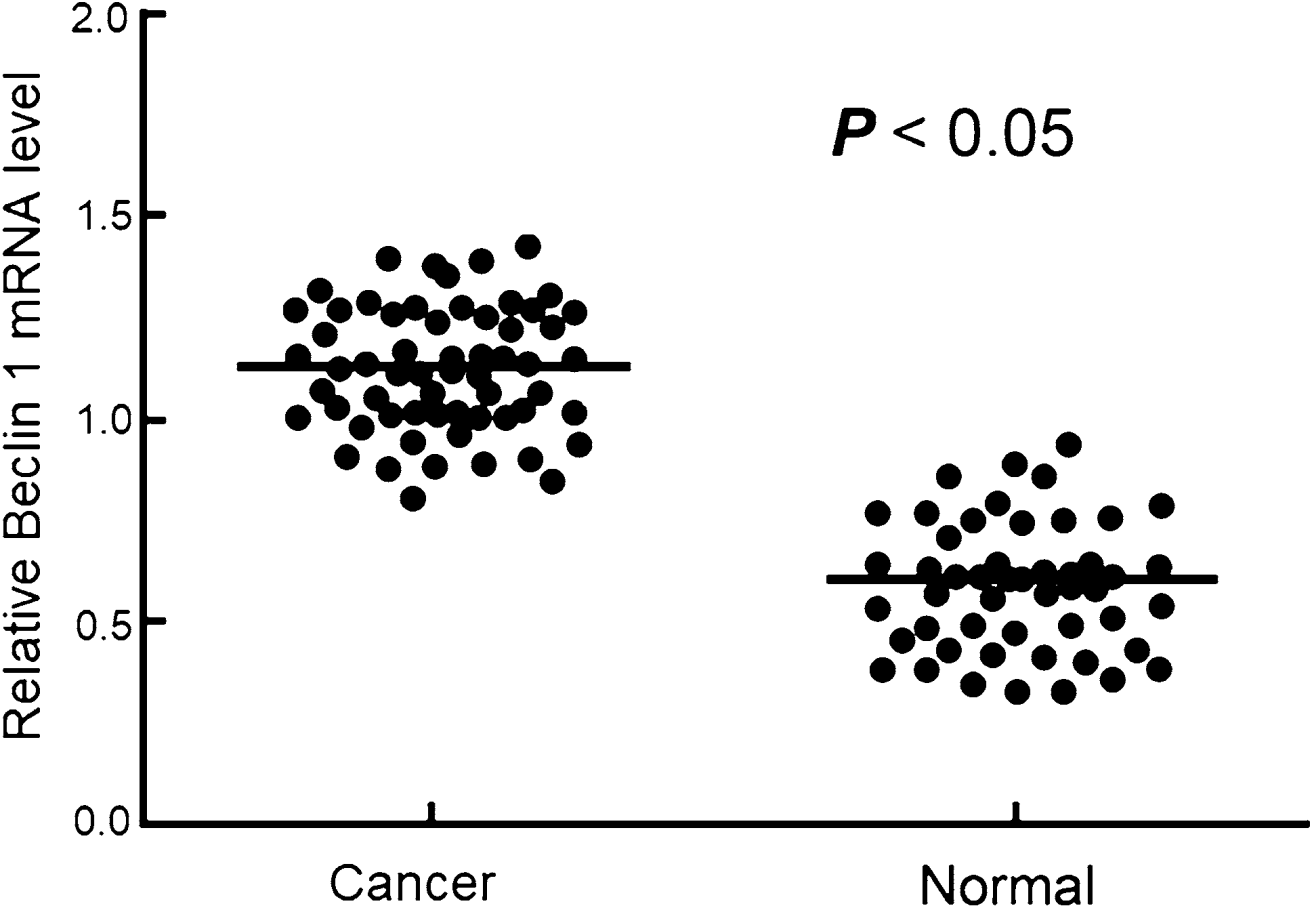
Relative mRNA level of Beclin 1 in patients with and without non-squamous non-small cell lung cancer determined by qRT-PCR

mRNA levels of Beclin 1 were compared in 50 tissue pairs of normal tumor adjacent and corresponding tumor tissue and the results showed that Beclin 1 mRNA levels were significantly higher in the tumor tissues than in the normal tumor adjacent tissues (*P* < 0.05, Fig. 1). In the cancer patient samples, the average relative expression level was 1.1. There were some overlaps in the expression levels between low cancer expressor and high non-cancer expressor (Fig. 1). When the average expression level was used as cutoff to group the patients, 235 patients were classified as high Beclin 1 expressors (Table 2). Assessments of EGFR and ALK mutations revealed that out of the 480 patients, 233 (48.5%) and 75 (12.6%) patients were positive for EGFR and ALK mutations, respectively (Table 2). Univariate analysis showed that Beclin 1 expression and EGFR and ALK mutations were associated with lymph node metastasis, TNM stage, tumor differentiation and prognosis, but not with gender, age and smoking status (Table 2, *P* < 0.05).

### Survival analysis

**Fig. 2:**
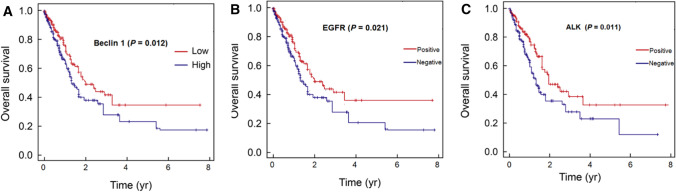
The Kaplan–Meier survival analysis of non-squamous non-small cell lung cancer patients with different Beclin 1 level, EGFR and ALK mutations

The Kaplan–Meier (KM) method was used to analyze time-to- all-cause mortality for patients with high and low Beclin 1 expression, and negative and positive EGFR mutations and ALK rearrangements. As shown in Fig. 2, high Beclin 1 expression and negative EGFR mutations and ALK rearrangements resulted in significantly lower survival rates as compared with low Beclin 1 expression and positive EGFR mutations and ALK rearrangements. The five-year survival rates in high Beclin 1 expression and negative EGFR mutations and ALK rearrangements vs low Beclin 1 expression and positive EGFR mutations and ALK rearrangements were 25.3% vs 45.3%, 22.3% vs 44.1% and 20.7% vs 40.1% (Fig. 2). The median progress-free survival (PFS) in the patients were 27.44 months. PFS of patients with low Beclin 1 expression and positive EGFR mutations and ALK rearrangements were significantly longer than those with high Beclin 1 expression and negative EGFR mutations and ALK rearrangements (Table 3, P < 0.000).

**Table 3:** Relationship between progress-free survival and Beclin 1 expression, *EGFR* and *ALK* rearrangements in lung cancer patients

Gene	Expression or mutation	Progress-free survival (month)			t	*P value*
		Average	Standard error	95% CI		
Beclin 1	High	22.11	3.21	17.87–34.21	4.882	0.000
	Low	34.91	4.61	27.27–47.11		
EGFR	Negative	18.33	5.11	12.17–24.66	7.542	0.000
	Positive	27.14	3.91	19.07–38.71		
*ALK*	Negative	21.31	4.01	16.17–31.41	11.324	0.000
	Positive	31.21	4.11	27.57–44.26		

### Risk factor and ROC analysis

To assess the factors associated with PFS, Beclin 1 level, EGFR and ALK mutations, TNM stage, differentiation and lymph node metastasis were included in the Cox regression models as independent variables. The results showed that Beclin 1 (OR = 2.882), EGFR (OR = 1.672), ALK (OR = 6.982) mutations and tumor differentiation grade (OR = 2.212), TNM stage (OR = 2.772) and lymph node metastasis (OR = 6.222) were independently associated with PFS in the patients with NSCLS (Table 4), and high Beclin 1 expression and negative EGFR and ALK mutations were associated with shorter PFS (Table 4).

**Table 4:** Multivariate survival analysis of factors associated with progress-free survival in lung cancer patients

Factors	β	SE	Wald *X*^2^	OR (95% CI)	*P* value
Beclin 1	1.022	0.432	5.675	2.882 (1.343–5.456)	0.000
EGFR	1.062	0.482	6.633	1.672 (1.133–3.444)	0.009
ALK	0.122	0.675	5.113	6.982 (3.641–8.443)	0.000
Tumor differentiation grade	2.062	0.657	1.985	2.212 (1.013–4.443)	0.016
TNM stage	0.421	0.223	1.689	2.772 (1.764–5.438)	0.012
Lymph node metastasis	1.762	0.897	11.625	6.222 (3.143–9.366)	0.000

**Fig. 3:**
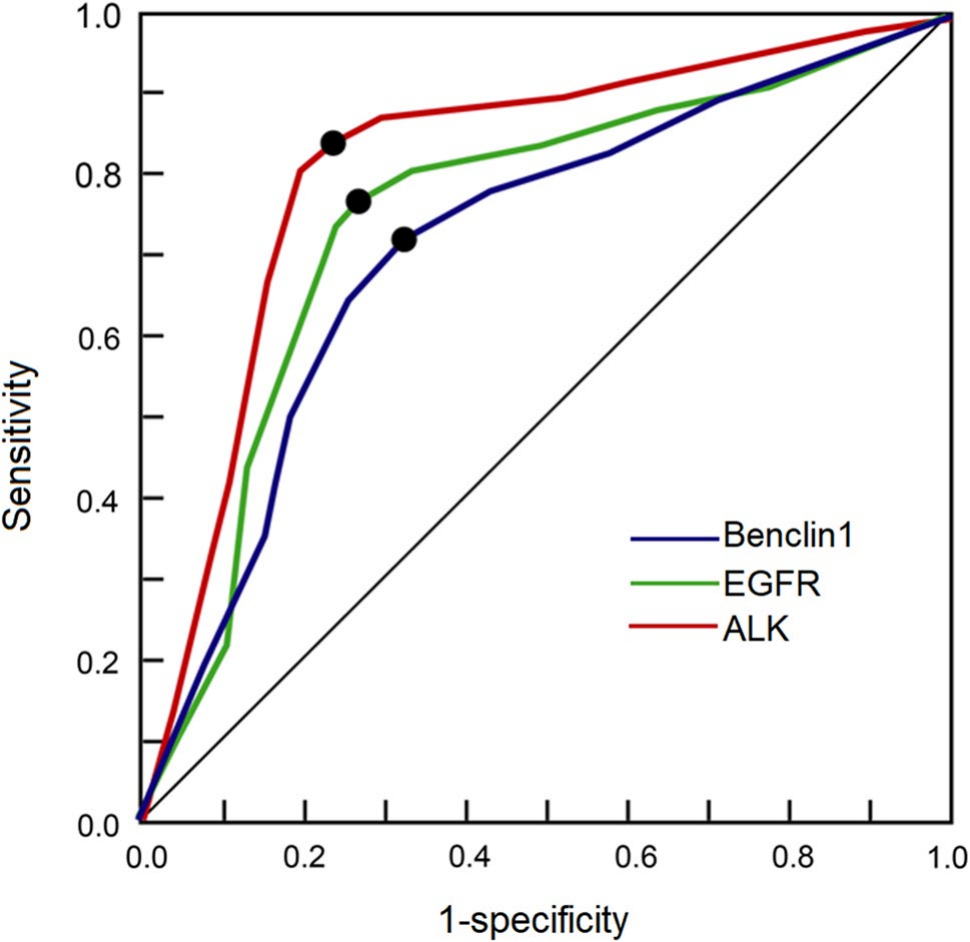
Receiver operating characteristic (ROC) curves of Beclin level, EGFR and ALK to predict progress-free survival time. The areas under curve (AUC) and cut-off values for Beclin 1, EGFR and ALK were 0.812 and 1.2, 0.781 and 15%, 0.722 and 11% respectively. Dots denote the Youden index

The ability of Beclin 1 expression, EGFR and ALK mutations to predict postoperative PFS in the patients with non-squamous NSCLC was studied using ROC curves and the AUCs. The results showed that these variables were significant predictors for PFS (Fig. 3, Table 5). The AUCs and cut-off values for Beclin 1, EGFR and ALK were 0.812 and 1.2 (*P* = 0.018), 0.781 and 15% (*P* = 0.011) and 0.722 and 11% (*P* = 0.010), respectively (Table 5).

**Table 5:** ROC analysis of Beclin 1 expression, *EGFR* and *ALK* mutations to predict postoperative PFS in non-squamous NSCLC patients

Variables	AUC	*P*	Cut-off value	Sensitivity (%)	Specificity (%)
Beclin 1	0.812	0.018	1.2	88.2	67.7
*EGFR*	*0.781*	0.011	15%	87.5	78.3
*ALK*	*0.722*	0.010	11%	89.2	80.1

## Discussion

Our study showed that Beclin 1 expression, EGFR and ALK mutations are associated with the prognosis of lung patients and have prognostic value to predict the survival of the patients. They may be used as standing alone biomarkers or in combination with other diagnostic tools to precisely predict the outcome of therapeutic plan.

Currently, the 5-year survival rate of NSCLC patients is only 15%. It is therefore very important to identify biological markers that can accurately evaluate the biological behavior of cancer (such as mutation, drug resistance, metastasis) and better predict the prognosis for developing targeted treatment plan and improve the efficiency of diagnosis and treatment and reduce the risk of death [33]. At present, there are various diagnostic and prognostic indicators for NSCLC, including serology indicators, such as carcinoembryonic antigen [34], CA-125 [35], cytokeratin 19 fragment 21–1 [36], neuron-specific enolase [37], histological indicators such as EGFR [38]. ALK [39], ROS-1 [40] and VEGF [41]. Serum markers are easy to be affected by many factors such as smoking, diet, infection, physiological state, leading to false negative or false positive results. On other hand. histopathological tests are more reliable and are the gold standard for the diagnosis of many diseases and can distinguish the morphological structures. However, for patients with non-squamous NSCLC, relatively less markers are available and more works are needed to demonstrate the clinical relevance and significance of the existing markers.

Beclin 1 is an autophagy-related gene and it interacts with Bcl-2 to induce apoptosis via binding to Bcl-2 and Bcl-xL, followed by the release of cytochrome c into the cytosol and activation of caspases [42]. The prognostic role of Beclin 1 in lung cancer is still controversial. Lee et al. showed that high Beclin1 expression predicts longer survival in locally advanced NSCLC [43], while Du et al. found that in patients with NSCLC, more advanced NSCLC was found to be associated with low Beclin-1 expression [44]. Although Beclin-1 expression is significantly associated with overall survival (OS), this association is only found in patients with high Bcl-2 expression, suggesting that Beclin1 may interact with other genes to exert its biological functions [44]. In this study, we found that the mRNA level of Beclin-1 is significantly elevated in cancer tissue samples as compared to adjacent normal tissue and non-squamous NSCLC patients with high Beclin-1 are associated with high mortality rates and shorter PFS. Previously, heavy smoking was found to be associated with Beclin 1 expression in patients with NSCLC [45], suggesting that Beclin 1 is likely to have a role to promote cancer progression, although Jiang et al. showed that Beclin 1 expression is not affected by smoking in lung cancer [46]. In this study, we did not see the association of Beclin 1 with smoking, but high Beclin 1 appeared to be related to lymph node metastasis and high TNM stage.

EGFR, as a member of the receptor tyrosine kinase family, is closely associated with the occurrence and development of NSCLC [38], ovarian cancer [47] and breast cancer [48]. EGFR tyrosine kinase inhibitors (such as gefitinib, erlotinib and afatinib) have been developed as the first-line and second-line targeting drags for NSCLC [49, 50]. However, the relationship between the expression of EGFR and the prognosis of patients is still controversial. Sonobe et al. found that EGFR gene mutations are not associated with the 5-year survival rate for all patients with completely resected pathological stage I-IIIA NSCLC, but the 5-year survival rate of patients with either a stage I adenocarcinoma or large cell carcinoma who had an EGFR mutation was significantly greater than those who did not have such a mutation [51], suggesting that EGFR mutation has different impact on different types of lung cancer. In this study, we found that the EGFR mutation is related to the prognosis and patients with EGFR mutations have significantly lower mortality and longer survival time as compared with patients with negative EGFR mutation. In addition, positive EGFR mutation appears to be associated with low risk of lymph node metastasis. In previous studies, it was found that EGFR mutation may result in the activation of the Ras-mitogen activated protein kinase (MAPK) pathway and PI3K/Akt signaling pathway, leading to increased cell proliferation, differentiation and angiogenesis and subsequently increased cell invasion and distant metastasis [52, 53]. For instance, as a result of EGFR activation, PI3K/AKT/mTOR signaling is active in over 90% of head and neck cancer^47^. In the non-squamous NSCLC, the role of EGFR mutations on of lymph node metastasis have not been fully investigated and more works are needed to elucidate the molecular mechanisms.

ALK gene rearrangements are present in a small subset of NSCLC [54]. In this study, we found that about 15.6% (75/480) patients had rearrangements, which appears to be relatively high. This may be attributed to the specific patient cohort (older patients) and the sensitivity of detection method. Previously, up to 7% EML4/ALK fusion rate was found in patients with brain metastases [55]. The ALK gene is often fused with EML4 gene in NSCLC, leading to continuous expression of ALK and the activation of downstream PI3K/Akt/MAPK signal pathway and occurrence of tumors [56, 57]. In this study, we found that ALK mutation is associated with pathological features of tumors (lymph node metastasis, TNM stage, differentiation) as well as mortality. Zhao et al. found that EML4-ALK fusion gene is significantly higher in patients with III-IV stage NSCLC than with I-II stage NSCLC [58] and in patients with brain metastases, the fusion may occur in 3–7% patients [55]. Gao et al. showed that patients with NSCLC have a positive rate of ALK up to 9.0% and between 2.4% and 8.8% in early stage patients [59], which is consistent with the results of this study that the positive rate is higher is in the early stage patients. The risk of lymph node metastasis in ALK positive patients was significantly lower than that in negative patients. Earlier study also showed that the OS time of ALK rearrangement positive patients was 97.7 months, significantly longer than that of ALK negative patients (78.9 month)[60], suggesting that ALK rearrangement positive patients have better prognosis, which is consistent with our results. It is likely that the signal pathway of downstream ALK is blocked after patients received targeted therapy with immune checkpoint inhibitors, leading to the suppression of growth and invasion of tumor cells and better therapeutic effect.

Our analysis also showed that Beclin 1, EGFR and ALK are independently associated with PFS and are significant predictors for PFS in the patient with non-squamous NSCLC with AUCs above 0.70, suggesting that they may be used as standing alone biomarkers or in combination with other diagnostic tools to precisely predict the outcome of therapeutic plan. Since these markers can be assessed using biopsy samples or surgically resected samples, the results could be available at the early stage of treatment and would be used to stratify patients for optimal chemotherapy options including the use of immune checkpoint inhibitors [61, 62].

There are several important limiting points in this study: it is its retrospective nature and single center-study with limited number of participants, patients were limited to elderly, were followed-up for relative short time and were not subjected to the same surgical treatments. Large and perspective studies are needed to further validate our conclusions.

## Conclusion

Non-squamous NSCLC is one of the most aggressive subtypes of lung cancer. Beclin 1 expression, EGFR and ALK mutations are independently associated with the prognosis and have prognostic value to predict PFS in the patient with non-squamous NSCLC. Therefore, Beclin 1, EGFR and ALK may be used as standing alone biomarkers or in combination with other diagnostic tools to predict the outcome of therapeutic plan and stratify patients for better prognosis.

## Data Availability

The datasets used and/or analysed during the current study are available from the corresponding author on reasonable request.

## Abbreviations

EGFR: Epidermal growth factor receptor
ALK: Anaplastic lymphoma kinase
NSCLC: Non-small cell lung cancer
PCR: Polymerase chain reaction
FISH: Fluorescent in situ hybridization
ROC: Receiver response curves
TNM: Tumor: node: metastasis
AUC: Areas under curve
qRT-PCR: Quantitative reverse transcription-polymerase chain reaction
ARMS: Amplification refractory mutation system
FFPE: Formalin-fixed paraffin-embedded
IQR: Interquartile range
SD: Standard deviation
OR: Odds ratios
CI: Confidence intervals
PFS: Progress-free survival
MAPK: Mitogen activated protein kinase
